# Supplementary material for: A non-specialist worker delivered digital assessment of cognitive development (DEEP) in young children: A longitudinal validation study in rural India
Source: PLOS Digit Health. 2025 May 16;4(5):e0000824. doi: 10.1371/journal.pdig.0000824 (PMC12084064; doi:10.1371/journal.pdig.0000824)
Supplement: S1 Text — (DOCX) [file pdig.0000824.s001.docx]

# **S1 Text: Detailed description of metrics derived from the DEEP tool.**

1. Accuracy: Computed by dividing the number of “correct” clicks or drags from the total number of clicks or drags within each game level, ranges from 0-1.
2. Highest_level: Most games have levels in which the difficulty of the game increases to capture variability in cognitive abilities (see Table 1 for a description of how difficulty is increased in each game). If a child does not successfully complete any difficulty level, the tool automatically terminates the game and presents the child with the next game. Odd One Out (OOO), Series Completion (SC) and Sorting Objects (SO) are designed to measure children’s ability in different dimensions like colour, size and numeracy and each dimension has 3 levels of difficulty. In these games, even if a child failed a difficulty level for one dimension (e.g. colour), they were presented with the next dimension (e.g. size). Highest_Level captures the number of difficulty levels played for each game and can range from 0-15. Exceptions for which this metric was not included are Location Recall (LR) which is woven into the storyline and so the presentation of game levels is not dependent on a child’s ability; Single Tap (ST) and Alternate Tap (AT) which only have 1 difficulty level; Popping Bubbles (PB) in which the presentation of the second difficulty level was not contingent on child performance on the first level.
3. Completion_time: Most games have time limits for completion of each level (see Table 1) such that if a child successfully completes the level within the time limit, the time of the last correct click or drag is recorded, the game level ends and the next difficulty level is presented while if they are unsuccessful within the set time limit, the tool does not present them with the next level but instead proceeds to the next game. Completion_time is the proportion of time within which the child completed the level, derived using the time of the last correct click divided by the time limit for that level, and ranges from 0-1. Exceptions for which this metric was not included are ST, AT and PB the child is presented with a screen on which they play for a fixed period of time; and Grow Your Garden (GYG) since the order in which target and distractor stimuli were presented was randomised implying that it could take longer to complete a level just due to the presentation of stimuli.
4. Latency: Represents the time taken for the first click or drag (could be either correct or incorrect) done by the child in every game level and ranges from 0 to the time limit of each game level.
5. Activity: Computed by dividing the total number of clicks or drags in a game level by Completion_time, except in ST, AT and PB in which they were divided by the game time limit. It represents the number of clicks or drags done by the child per second and ranges from 0.01 to 50.
