# Supplementary material for: A non-specialist worker delivered digital assessment of cognitive development (DEEP) in young children: A longitudinal validation study in rural India
Source: PLOS Digit Health. 2025 May 16;4(5):e0000824. doi: 10.1371/journal.pdig.0000824 (PMC12084064; doi:10.1371/journal.pdig.0000824)
Supplement: S2 Text — (DOCX) [file pdig.0000824.s002.docx]

# **S2 Text: Details of the item-response theory modelling**

The Graded Response Model (GRM) is a widely used item response theory model designed for ordered polytomous response categories. The GRM assumes that each item response can be described by a series of cumulative probabilities corresponding to different difficulty thresholds, which define the boundaries between adjacent response categories. The probability of obtaining $x_{j}$, a given difficulty threshold for item $j$, or higher is given by:

$$P_{x_{j}}^{*}\left( \theta\right)=\frac{e^{\alpha_{j}\left( \theta-\delta_{x_{j}} \right)}}{1+e^{\alpha_{j}\left( \theta-\delta_{x_{j}} \right)}}$$

Where $\theta$ is ability, $\alpha_{j}$ is the discrimination for item $j$, and $\delta_{x_{j}}$ is the difficulty location or threshold for category $x$ for item $j$. Discrimination indicates and item’s ability to differentiate between individuals with different levels of ability. Functionally, discrimination parameters that are too low indicate the item is not useful for measurement of the ability trait while those that are too high (a rare account) indicate the item is only measuring a very specific region of the ability trait. Difficulty indicates the level of ability required to have a 50% chance of answering with a given ordinal category or higher. In practice, we want to use a range of items whose difficulty locations cover the range of abilities we expect to measure.

Data from four of the five metrics, Accuracy, Completion_time, Latency and Activity, was continuous in nature and non-normally distributed. Initially, mixed effect generalised linear regression models were intended to be used to model the scores. However, due to the complexity of jointly modelling the differing implied error distributions (i.e., proportions, time to event), it was decided to segment the metrics so each metric would be distributed as ordinal and IRT analysis could be used to accommodate all metrics. For the metrics Accuracy and Completion_time, data was categorized into equal intervals e.g.., >=0 to <=0.33, >0.33 to <=0.66, and >0.66 to <=1. For Latency and Activity, data was divided into categories, based on terciles in the whole sample. Segmenting the data into 5 categories was not found to improve performance of models derived from single variables and so all subsequent modelling was done using 3 categories (S1 Table). Further, models using only Latency and Activity data demonstrated poor prediction and were thus excluded from further analysis, and various combinations of the remaining three variables were tested. The final model chosen used three metrics: Accuracy, Highest_level and Completion_time.

The discrimination of the metrics of every game, averaged across game levels, and the difficulty for each game level, averaged across response options, were computed (S2 Table). The Highest_level metrics for all games and Accuracy and Completion_time for MS, JIG, SO and SD games show high discriminations. Overall, a trend in average game difficulty is observed such that the games which appear first in the tool are easier than those that appear later. However, items did not uniformly increase in difficulty across levels in all games. Test reliability was >0.90 between DEEP scores of 5-75 (S1A Figure). The standard error of estimation (SEE) demonstrated the effect of the additional games on increasing the precision of the tool which can be seen when we compare the circles (younger) to the triangles (older) samples (S1B Figure).
