# Supplementary material for: A non-specialist worker delivered digital assessment of cognitive development (DEEP) in young children: A longitudinal validation study in rural India
Source: PLOS Digit Health. 2025 May 16;4(5):e0000824. doi: 10.1371/journal.pdig.0000824 (PMC12084064; doi:10.1371/journal.pdig.0000824)
Supplement: S1 Table — (DOCX) [file pdig.0000824.s003.docx]

# **S1 Table: Models explored to derive DEEP-score.**

Variable combinations used, number of categories into which continuous data was segmented, correlation with age and model fit indices across explored models. The final chosen model (S.No. 10) is in bold.

| **S.No.** | **Model** | **Variables** | **Categories** | **Correlation with age**  **r (95% CI)** | **RMSEA** | **TLI** | **CFI** |
| --- | --- | --- | --- | --- | --- | --- | --- |
| Single variables | | | | | | | |
| 1 | Highest_level | 1 | 0-15 | 0.87 (0.86 – 0.89) *** | 0.04 | 0.99 | 1.00 |
| 2 | Accuracy | 1 | 3 | 0.78 (0.76 – 0.79) *** | 0.02 | 0.99 | 0.99 |
| 3 | Accuracy |  | 5 | 0.79 (0.78 – 0.80) *** | 0.03 | 0.99 | 0.99 |
| 4 | Completion_time | 1 | 3 | 0.69 (0.67 – 0.71) *** | 0.02 | 1.00 | 1.00 |
| 5 | Completion_time |  | 5 | 0.70 (0.68 – 0.72) *** | 0.02 | 0.99 | 0.99 |
| 6 | Latency | 1 | 3 | 0.08 (0.04 – 0.11) *** | 0.03 | 0.88 | 0.88 |
| 7 | Latency |  | 5 | 0.03 (-0.01 – 0.07) **ns** | 0.04 | 0.9 | 0.9 |
| 8 | Activity | 1 | 3 | 0.13 (0.09 – 0.17) *** | 0.04 | 0.91 | 0.91 |
| 9 | Activity |  | 5 | 0.23 (0.19 – 0.27) *** | 0.05 | 0.91 | 0.91 |
| Combining variables | | | | | | | |
| **10** | **Highest_level + Accuracy + Completion_time** | **3** | **3** | **0.83 (0.82 – 0.84) ***** | **0.03** | **0.99** | **0.99** |
| 11 | Accuracy + Completion_time | 2 | 3 | 0.78 (0.76 – 0.79) *** | 0.03 | 0.99 | 0.99 |
| 12 | Highest_level + Accuracy | 2 | 3 | 0.85 (0.84 – 0.86) *** | 0.03 | 0.99 | 0.99 |
| 13 | Highest_level + Completion_time | 2 | 3 | 0.82 (0.81 – 0.83) *** | 0.02 | 0.99 | 0.99 |

RMSEA = root mean square error of approximation, TLI = Tucker-Lewis Index, CFI = Comparative Fit Index (CFI); *<0.05; **<0.01; ***<0.001; ns ≥ 0.05
