# Supplementary material for: A non-specialist worker delivered digital assessment of cognitive development (DEEP) in young children: A longitudinal validation study in rural India
Source: PLOS Digit Health. 2025 May 16;4(5):e0000824. doi: 10.1371/journal.pdig.0000824 (PMC12084064; doi:10.1371/journal.pdig.0000824)
Supplement: S2 Table — (DOCX) [file pdig.0000824.s004.docx]

# **S2 Table: Discrimination and difficulty of DEEP items**

The discrimination of the metrics of every game, averaged across game levels, and the difficulty for each game level, averaged across response options (grey colour = not applicable) for the final chosen model.

|  | **Game** | **Avg. Discrimination** | **Avg. Difficulty** | **L1** | **L2** | **L3** | **L4** | **L5** | **L6** | **L7** | **L8** | **L9** | **L10** | **L11** | **L12** | **L13** | **L14** | **L15** |
| --- | --- | --- | --- | --- | --- | --- | --- | --- | --- | --- | --- | --- | --- | --- | --- | --- | --- | --- |
| **Highest_level** | LR |  |  |  |  |  |  |  |  |  |  |  |  |  |  |  |  |  |
|  | ST |  |  |  |  |  |  |  |  |  |  |  |  |  |  |  |  |  |
|  | AT |  |  |  |  |  |  |  |  |  |  |  |  |  |  |  |  |  |
|  | PB |  |  |  |  |  |  |  |  |  |  |  |  |  |  |  |  |  |
|  | GYG | 1.73 | -0.89 |  |  |  |  |  |  |  |  |  |  |  |  |  |  |  |
|  | HO | 2.54 | -1.20 |  |  |  |  |  |  |  |  |  |  |  |  |  |  |  |
|  | OOO | 3.10 | -1.04 |  |  |  |  |  |  |  |  |  |  |  |  |  |  |  |
|  | SD | 2.28 | -0.89 |  |  |  |  |  |  |  |  |  |  |  |  |  |  |  |
|  | MS | 5.06 | -0.30 |  |  |  |  |  |  |  |  |  |  |  |  |  |  |  |
|  | JIG | 4.12 | -0.39 |  |  |  |  |  |  |  |  |  |  |  |  |  |  |  |
|  | SO | 3.34 | -0.54 |  |  |  |  |  |  |  |  |  |  |  |  |  |  |  |
|  | SC | 2.86 | -0.07 |  |  |  |  |  |  |  |  |  |  |  |  |  |  |  |
|  | PM | 2.61 | 0.84 |  |  |  |  |  |  |  |  |  |  |  |  |  |  |  |
|  | SR | 2.03 | 0.48 |  |  |  |  |  |  |  |  |  |  |  |  |  |  |  |
| **Accuracy** | LR | 0.91 | -1.13 | -1.84 | -1.64 | -1.12 | -0.88 | -0.22 | -0.95 | -1.18 | -1.24 |  |  |  |  |  |  |  |
|  | ST | 0.89 | -3.99 | -3.99 |  |  |  |  |  |  |  |  |  |  |  |  |  |  |
|  | AT | 2.26 | -1.40 | -1.40 |  |  |  |  |  |  |  |  |  |  |  |  |  |  |
|  | PB | 1.58 | -1.78 | -1.90 | -1.67 |  |  |  |  |  |  |  |  |  |  |  |  |  |
|  | GYG | 1.29 | -1.50 | -2.10 | -1.76 | -2.29 | 1.12 | -2.51 |  |  |  |  |  |  |  |  |  |  |
|  | HO | 1.42 | -1.59 | -2.16 | -1.38 | -0.70 | -0.73 | -0.61 | -3.17 | -2.37 |  |  |  |  |  |  |  |  |
|  | OOO | 0.81 | -0.13 | -0.68 | -1.27 | -0.62 | -0.13 | 0.15 | -0.43 | -0.93 | -0.56 | 0.58 | -1.46 | 1.51 | 0.88 | 0.33 | 0.03 | 0.67 |
|  | SD | 1.49 | -0.22 | -0.57 | 0.08 | -0.15 |  |  |  |  |  |  |  |  |  |  |  |  |
|  | MS | 2.16 | -0.40 | -0.51 | -0.82 | -0.87 | -0.42 | -0.25 | -0.43 | 0.75 | -0.36 | -0.69 |  |  |  |  |  |  |
|  | JIG | 1.59 | -0.33 | -0.85 | -1.12 | -0.69 | -0.49 | -0.48 | -0.56 | 0.56 | 0.69 | -0.01 |  |  |  |  |  |  |
|  | SO | 1.48 | -0.98 | -2.86 | -0.46 | -0.73 | -0.32 | -1.07 | -1.30 | -0.72 | -0.44 | -0.87 |  |  |  |  |  |  |
|  | SC | 0.91 | 0.46 | 1.24 | 0.69 | 1.09 | 0.68 | 0.75 | -2.56 | 1.21 | 0.06 | 0.99 |  |  |  |  |  |  |
|  | PM | 0.76 | 0.48 | 0.85 | -2.33 | 2.92 |  |  |  |  |  |  |  |  |  |  |  |  |
|  | SR | 0.94 | 1.12 | -0.58 | 1.08 | 2.86 |  |  |  |  |  |  |  |  |  |  |  |  |
| **Completion_time** | LR | 1.15 | -4.11 | -6.37 | -5.08 | -4.15 | -3.12 | -3.28 | -3.62 | -3.57 | -3.72 |  |  |  |  |  |  |  |
|  | ST |  |  |  |  |  |  |  |  |  |  |  |  |  |  |  |  |  |
|  | AT |  |  |  |  |  |  |  |  |  |  |  |  |  |  |  |  |  |
|  | PB |  |  |  |  |  |  |  |  |  |  |  |  |  |  |  |  |  |
|  | GYG |  |  |  |  |  |  |  |  |  |  |  |  |  |  |  |  |  |
|  | HO | 1.06 | -1.50 | -3.52 | -2.33 | -1.26 | -1.59 | -1.02 | -1.28 | 0.48 |  |  |  |  |  |  |  |  |
|  | OOO | 1.08 | -4.07 | -2.31 | -2.71 | -3.90 | -1.92 | -1.09 | -2.65 | -2.45 | -2.06 | -29.17 | -3.61 | -3.53 | -1.99 | -0.89 | -1.45 | -1.42 |
|  | SD | 1.98 | -0.97 | -1.31 | -0.48 | -1.12 |  |  |  |  |  |  |  |  |  |  |  |  |
|  | MS | 3.11 | -0.47 | -0.70 | -0.39 | -1.01 | -0.73 | -0.57 | -0.31 | 0.07 | -0.29 | -0.32 |  |  |  |  |  |  |
|  | JIG | 2.07 | -0.34 | -0.94 | -1.13 | -0.91 | -1.05 | -0.66 | -0.51 | 1.02 | 0.78 | 0.32 |  |  |  |  |  |  |
|  | SO | 2.50 | -0.65 | -1.12 | -0.39 | -0.54 | -0.22 | -0.81 | -0.96 | -0.83 | -0.34 | -0.62 |  |  |  |  |  |  |
|  | SC | 1.18 | -0.64 | 0.96 | 0.31 | -0.32 | 0.39 | -0.09 | -2.99 | 0.29 | -2.05 | -2.27 |  |  |  |  |  |  |
|  | PM | 0.93 | 0.22 | 0.86 | 1.52 | -1.73 |  |  |  |  |  |  |  |  |  |  |  |  |
|  | SR | 1.37 | -0.85 | -1.64 | -0.45 | -0.46 |  |  |  |  |  |  |  |  |  |  |  |  |
