# Supplementary material for: A non-specialist worker delivered digital assessment of cognitive development (DEEP) in young children: A longitudinal validation study in rural India
Source: PLOS Digit Health. 2025 May 16;4(5):e0000824. doi: 10.1371/journal.pdig.0000824 (PMC12084064; doi:10.1371/journal.pdig.0000824)
Supplement: S3 Table — (DOCX) [file pdig.0000824.s005.docx]

# **S3 Table: Comparison of associations of criterion and predictive validity measures with ML-derived and IRT-derived DEEP scores.**

Associations of ML-derived and IRT-derived DEEP-score with BSID-III cognitive domain raw score, ASER Language, ASER Numeracy and ASER Cognitive Development.

| **Measure** | **Age of measurement** | **Age of DEEP measurement** | **Correlation**  **r, 95% CI(n)** | |
| --- | --- | --- | --- | --- |
|  |  |  | **ML-derived DEEP score** | **IRT-derived DEEP score** |
| **Criterion validity** | | | | |
| BSID-III cognitive domain raw score | 3-years (BL) | 3-years (BL) | 0.67***, 0.59-0.74(200) | 0.50***, 0.39-0.60(200) |
| **Predictive validity** | | | | |
| ASER Language | 8-years (FU2) | 3-years (BL) | 0.23***, 0.15 – 0.31(601) | 0.26***, 0.18 – 0.33(601) |
|  |  | 8-years (FU2) | NA | 0.40***, 0.34 – 0.47(600) |
| ASER Numeracy |  | 3-years (BL) | 0.34***, 0.27 – 0.41(601) | 0.32***, 0.25 – 0.39(601) |
|  |  | 8-years (FU2) | NA | 0.50***, 0.43 – 0.55(600) |
| ASER Cognitive Development^#^ |  | 3-years (BL) | 0.28***, 0.20 – 0.35(601) | 0.30***, 0.23 – 0.37(601) |
|  |  | 8-years (FU2) | NA | 0.43***, 0.36 – 0.50(600) |

*<0.05; **<0.01; ***<0.001; # Spearman’s correlation
