# Supplementary material for: A non-specialist worker delivered digital assessment of cognitive development (DEEP) in young children: A longitudinal validation study in rural India
Source: PLOS Digit Health. 2025 May 16;4(5):e0000824. doi: 10.1371/journal.pdig.0000824 (PMC12084064; doi:10.1371/journal.pdig.0000824)
Supplement: S4 Table — (DOCX) [file pdig.0000824.s006.docx]

# **S4 Table: Correlations between Annual Status of Education Report (ASER) and measures used for DEEP’s convergent validity.**

Associations of DEEP-score, ASER Language and ASER Numeracy with HAZ and early life adversities.

| **Measure, age** | **DEEP-score r, 95% CI (n)** | **ASER Language r, 95% CI (n)** | **ASER Numeracy r, 95% CI (n)** | **ASER Cognitive Development r, 95% CI (n)^#^** |
| --- | --- | --- | --- | --- |
| Height-for-age z score (HAZ), 8-years (FU 2) | 0.18***, 0.10 - 0.26 (600) | 0.21***, 0.13 - 0.28 (601) | 0.26***, 0.19 - 0.34 (601) | 0.22***, 0.14 – 0.29  (600) |
| Socioeconomic status quantile, Birth  (SPRING study data)^#^ | 0.27***, 0.19 – 0.35  (600) | 0.31***, 0.23 – 0.38  (601) | 0.31***, 0.23 – 0.38  (601) | 0.18***, 0.10 – 0.25  (601) |
| Child domain, 12-months (SPRING study data) | -0.03, -0.11 - 0.05 (600) | -0.08*, -0.16 - 0.00 (601) | -0.11**, -0.19 - -0.03 (601) | -0.10*, -0.18 – -0.02  (601) |
| Maternal stress domain, 12-months (SPRING study data) | -0.10, -0.18 - -0.02 (600) | -0.14**, -0.22 - -0.06 (601) | -0.13**, -0.21 - -0.05 (601) | -0.09*, -0.17 – -0.01  (601) |
| SES domain, 12-months (SPRING study data) | -0.25*, -0.32 - -0.17 (600) | -0.32***, -0.39 - -0.24 (601) | -0.31***, -0.38 - -0.24 (601) | -0.13**, -0.21 – -0.04  (601) |
| Relationship domain, 12-months (SPRING study data) | -0.07***, -0.16 - 0.03 (410) | -0.04, -0.14 - 0.06 (411) | -0.12*, -0.22 - -0.03 (411) | -0.07, -0.17 – 0.03  (411) |
| Cumulative adversity: 3 domains (without relationship domain), 12-months (SPRING study data) | -0.21***, -0.28 - -0.13 (600) | -0.29***, -0.36 - -0.21 (601) | -0.30***, -0.37 - -0.22 (601) | -0.16***, -0.23 – -0.08  (601) |
| Cumulative adversity: all domains, 12-months (SPRING study data) | -0.25***, -0.33 - -0.15 (410) | -0.30***, -0.38 - -0.21 (411) | -0.35***, -0.44 - -0.27 (411) | -0.18***, -0.27 – -0.09  (411) |

*<0.05; **<0.01; ***<0.001; # Spearman’s correlation
