# Supplementary material for: A non-specialist worker delivered digital assessment of cognitive development (DEEP) in young children: A longitudinal validation study in rural India
Source: PLOS Digit Health. 2025 May 16;4(5):e0000824. doi: 10.1371/journal.pdig.0000824 (PMC12084064; doi:10.1371/journal.pdig.0000824)
Supplement: S5 Table — (DOCX) [file pdig.0000824.s007.docx]

# **S5 Table: Correlations between Raven’s Coloured Progressive Matrices (CPM) and measures used for DEEP’s convergent validity.**

Associations of DEEP-score and CPM scores with HAZ and early life adversities.

| **Measure, age** | **DEEP-score r, 95% CI (n)** | **CPM set A r, 95% CI (n)** | **CPM set Ab r, 95% CI (n)** | **CPM set B r, 95% CI (n)** | **CPM Total r, 95% CI (n)** |
| --- | --- | --- | --- | --- | --- |
| Height-for-age z score (HAZ), 8-years (FU 2) | 0.18***, 0.10 - 0.26 (600) | 0.14***, 0.06 - 0.21 (599) | 0.14***(0.06 - 0.22 (599) | 0.13**, 0.05 - 0.21 (599) | 0.17***, 0.09 - 0.25 (599) |
| Socioeconomic status quantile, Birth  (SPRING study data)^#^ | 0.27***, 0.19 – 0.35  (600) | 0.16***, 0.07 – 0.24  (600) | 0.21***, 0.13 – 0.29  (600) | 0.23***, 0.15 – 0.31  (600) | 0.26***, 0.19 – 0.34  (599) |
| Child domain, 12-months (SPRING study data) | -0.03, -0.11 - 0.05 (600) | -0.03, -0.11 - 0.05 (599) | -0.03, -0.11 - 0.05 (599) | -0.05, -0.13 - 0.03 (599) | -0.05, -0.13 - 0.03 (599) |
| Maternal stress domain, 12-months (SPRING study data) | -0.10, -0.18 - -0.02 (600) | -0.11*, -0.20 - -0.01 (409) | -0.17***, -0.26 - -0.07 (409) | -0.11*, -0.21 - -0.02 (409) | -0.20***, -0.29 - -0.11 (409) |
| SES domain, 12-months (SPRING study data) | -0.25*, -0.32 - -0.17 (600) | -0.09*, -0.17 - -0.01 (599) | -0.09*, -0.17 - -0.01 (599) | -0.06, -0.14 - 0.02 (599) | -0.11**, -0.19 - -0.03 (599) |
| Relationship domain, 12-months (SPRING study data) | -0.07***, -0.16 - 0.03 (410) | -0.12**, -0.20 - -0.04 (599) | -0.15***, -0.23 - -0.08 (599) | -0.18***, -0.26 - -0.10 (599) | -0.21***, -0.28 - -0.13 (599) |
| Cumulative adversity: 3 domains (without relationship domain), 12-months (SPRING study data) | -0.21***, -0.28 - -0.13 (600) | -0.12**, -0.20 - -0.05 (600) | -0.14***, -0.22 - -0.06 (600) | -0.16***, -0.23 - -0.08 (600) | -0.20***, -0.27 - -0.12 (599) |
| Cumulative adversity: all domains, 12-months (SPRING study data) | -0.25***, -0.33 - -0.15 (410) | -0.16**, -0.25 - -0.07 (409) | -0.22***, -0.31 - -0.12 (409) | -0.21***, -0.30 - -0.12 (409) | -0.28***, -0.37 - -0.19 (409) |

*<0.05; **<0.01; ***<0.001; # Spearman’s correlation
