# Supplementary material for: A non-specialist worker delivered digital assessment of cognitive development (DEEP) in young children: A longitudinal validation study in rural India
Source: PLOS Digit Health. 2025 May 16;4(5):e0000824. doi: 10.1371/journal.pdig.0000824 (PMC12084064; doi:10.1371/journal.pdig.0000824)
Supplement: S1 Fig — (DOCX) [file pdig.0000824.s008.docx]

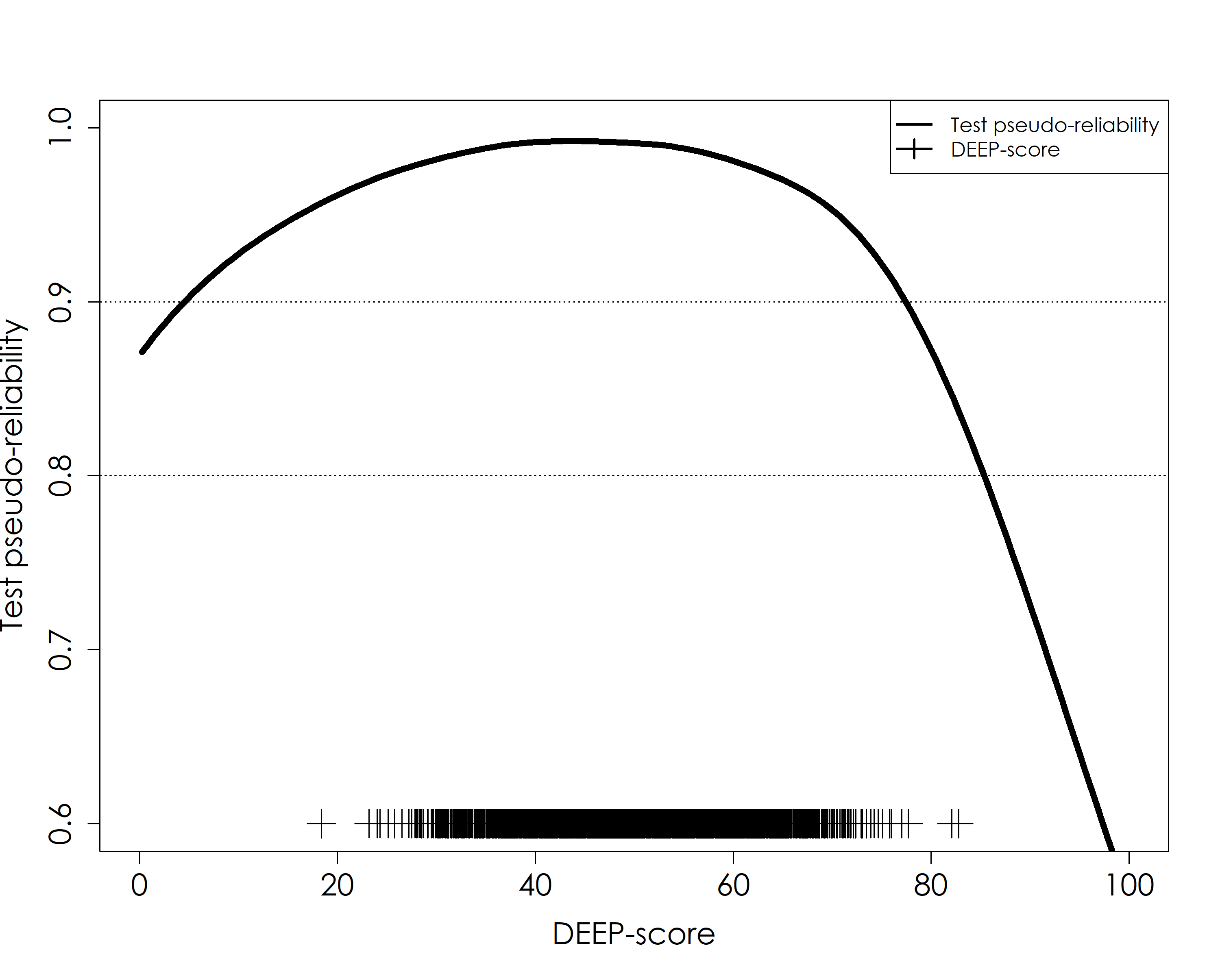


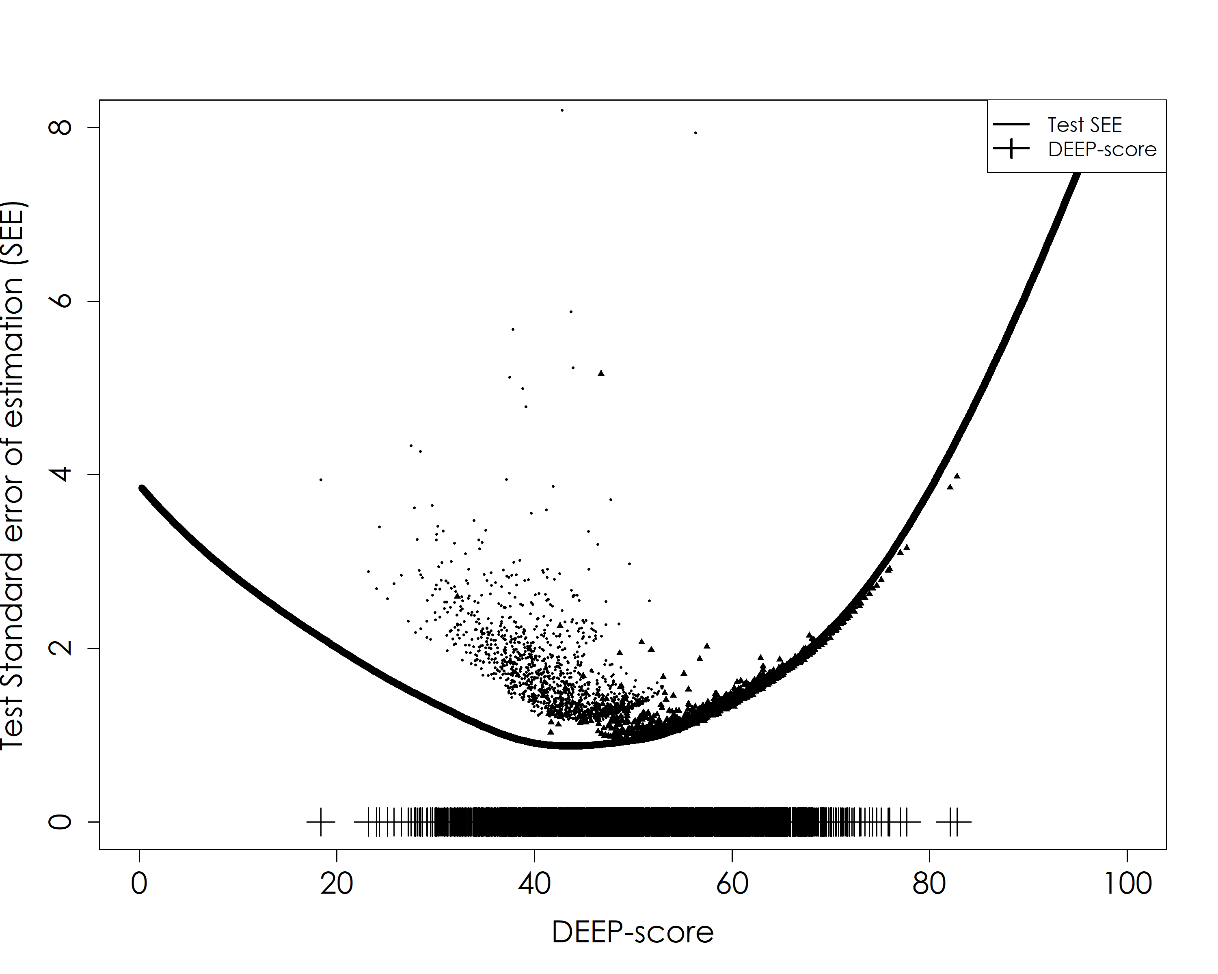


**S1 Figure: Test pseudo-reliability and standard error of estimation of the final model for DEEP-score.** (A) Test pseudo-reliability of DEEP-scores; (B) The Standard Error of Estimation (SEE) for younger (circles) and older (triangles) children.
